# Landscape Genomics in Tree Conservation Under a Changing Environment

**DOI:** 10.3389/fpls.2022.822217

**Published:** 2022-02-24

**Authors:** Li Feng, Fang K. Du

**Affiliations:** ^1^School of Pharmacy, Xi’an Jiaotong University, Xi’an, China; ^2^School of Ecology and Nature Conservation, Beijing Forestry University, Beijing, China

**Keywords:** changing environment, genotype-environment associations (GEAs), landscape genomics, local adaptation, tree conservation

## Abstract

Understanding the genetic basis of how species respond to changing environments is essential to the conservation of species. However, the molecular mechanisms of adaptation remain largely unknown for long-lived tree species which always have large population sizes, long generation time, and extensive gene flow. Recent advances in landscape genomics can reveal the signals of adaptive selection linking genetic variations and landscape characteristics and therefore have created novel insights into tree conservation strategies. In this review article, we first summarized the methods of landscape genomics used in tree conservation and elucidated the advantages and disadvantages of these methods. We then highlighted the newly developed method “Risk of Non-adaptedness,” which can predict the genetic offset or genomic vulnerability of species *via* allele frequency change under multiple scenarios of climate change. Finally, we provided prospects concerning how our introduced approaches of landscape genomics can assist policymaking and improve the existing conservation strategies for tree species under the ongoing global changes.

## Introduction

Forest trees cover *ca.* 30% of the terrestrial surface of the earth from boreal to tropical latitudes and contain approximately three-quarters of the terrestrial biomass of the earth, which tightly links them with the global carbon cycle (Holliday et al., 2017; Isabel et al., 2020). They generally have higher levels of genetic diversity and experience rapid microevolution, which often show distinguishable adaptation to local environments (Hamrick et al., 1992; Petit and Hampe, 2006; Neale and Kremer, 2011). In addition, quantitative traits with high heritability make trees exhibit stronger signals of local adaptation (clinal variation); however, a large genome, long generation time makes it not suitable for quantitative trait loci (QTL) and related analysis even though great progress have been achieved on quantitative genetics study on trees (Savolainen et al., 2013; Milesi et al., 2019 and references therein). Therefore, understanding the genetic basis of adaptation to the environment *via* landscape genomics studies is essential for management interventions of tree species related to conservation and reforestation under climate change (Allendorf et al., 2010; Savolainen et al., 2013; Anderson and Song, 2020).

Empirical studies had already suggested that adaptation in tree species primarily arises from standing genetic variations, facilitating more rapid adaptation to climate change than that *via* new mutations (Barrett and Schluter, 2008; Alberto et al., 2013; Savolainen et al., 2013). However, rapid climate change can break this association and create a mismatch between population climatic optima and current climate (Jump and Peñuelas, 2005; Aitken et al., 2008). Additional challenges such as gene flow, eco-evolutionary dynamics on the species range margins, and variation in climate changes across the landscape may also impact the adaptation of species (Savolainen et al., 2007; Alberto et al., 2013; Aitken and Bemmels, 2016). Still, in practice, very few conservation strategies consider genetic resources, especially for forest species (Lefèvre et al., 2013), with some applications in forest restoration program, e.g., it is recommended that *Quercus mongolica* seeds should not be transferred from their provenances because the genetic cline was determined between the northeastern and southwestern Japan by neutral genetic markers (Nagamitsu and Shuri, 2021).

Great progress had been achieved on the topic of the adaptive potential of natural populations (Hoffmann et al., 2017; Gaitán-Espitia and Hobday, 2021). In recent years, integrated interdisciplinary methods such as landscape genetics or genomics which are used to disentangle the impacts of environmental conditions on forest trees might provide guides for forest conservations (Rellstab et al., 2015; Isabel et al., 2020). The classical method for detecting the genetic basis of adaptation relies on population genetics (Wright and Gaut, 2004). This method attempts to find out outlier single-nucleotide polymorphisms (SNPs) by the comparisons of the genetic differentiation (*F*_ST_) between populations and to hypothesize that these outliers are most likely to be affected by natural selection (Excoffier et al., 2009; Hohenlohe et al., 2010). However, this method suffers a high ratio of false-positive results due to the ignorance of environmental heterogeneity (Eveno et al., 2008; Hoban et al., 2016). Another approach is landscape genomics, which uncovers the molecular mechanism of adaptation on the basis of the genotype-environment associations (GEAs) by integrating genetic variation and spatial models (Holderegger et al., 2006; Sork et al., 2013; Sork, 2017; De Lafontaine et al., 2018). Recently, the evaluation of the genomic vulnerability (Bay et al., 2018), genetic offset (Fitzpatrick and Keller, 2015), or risk of non-adaptedness (RONA) (Rellstab et al., 2016) was used to predict the climate-driven population shifts. At present, a growing number of studies focus on tree species already utilized the population-level genomic data to evaluate the genomic vulnerability of the species in a changing climate.

The next-generation sequencing makes landscape genomic studies currently possible for detecting adaptive signals and uncovering the genomic basis of adaptation in many organisms. Although landscape genomics has been pursued for a decade and the advances of theoretical frameworks and applications are promising (Schoville et al., 2012; Joost et al., 2013; Bragg et al., 2015; Ćalić et al., 2015; Rellstab et al., 2015; Capblancq et al., 2020), molecular ecologists and evolutionists are currently awash with data, and the analytical methods in landscape genomics have lagged behind.

**FIGURE 1:**
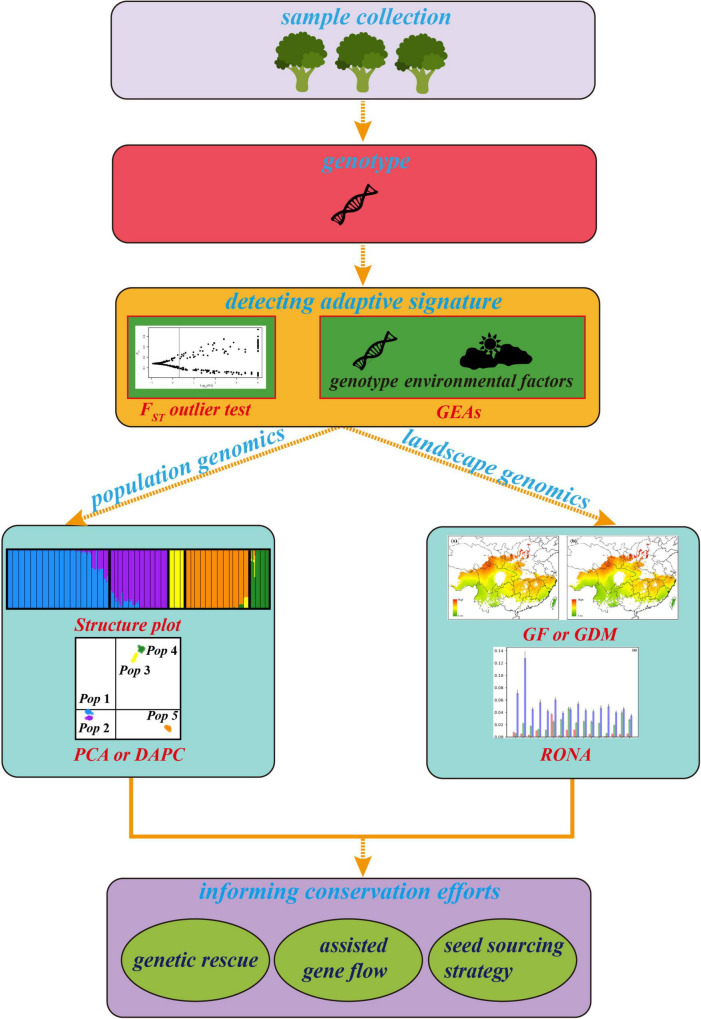
The general framework of landscape genomics for tree conservation. The plots of cluster, *F*_ST_ outlier test and RONA are modified from Du et al. (2020) and Feng et al. (2020), respectively.

As an emerging approach used for the conservation genetics of trees, it is essential to understand its advanced trend. However, the existing approaches belong to landscape genomics for detecting adaptive signatures and predicting genetic offset of adaptive allelic frequencies under multiple climates have different assumptions, advantages, and limitations. An effective integrative framework shortage and how to utilize the results from these variable methods to improve management interventions of forest trees are big challenges for landscape genomics studies in the genomic era. Therefore, we first surveyed recent literature on the landscape genomics approach used for tree conservation study. We checked the Original Journal articles in the *Molecular Ecology*, *Evolutionary Applications*, *Global Change Biology*, *New Phytologist*, *Ecology Letters*, and *Nature Climate Change* from 2015 to 2021 (Table 1). Publications were selected based on four criteria: (i) the research was performed on forest tree species; (ii) an SNP dataset was used; (iii) adaptive SNPs were detected, and (iv) articles must predict the optimal composition to the future climate to evaluate genetic offset. Second, we summarized and depicted the advantages and disadvantages of utilizing related methods and genomic tools involved in detecting GEAs (Table 2) to quantify and/or map the disruption in local adaptation of forest trees under climate change. Then, we established a general framework (Figure 1) integrated methods of landscape genomics and population genomics for local adaptation analysis in forest trees. Finally, we provided suggestions on how these approaches can be used in making conservation strategies for tree species under climate change.

**TABLE 1:** Short overview of recent studies of landscape genomics for forest trees.

Species	Spatial scale	Data	Adaptive signature identification	Predictive model	References	Journal
*Populus balsamifera*	North America	Targeted genotyping	*F*_ST_ outlier tests, Bayenv, GPA	GF, GDM	Fitzpatrick and Keller, 2015	Ecology letters
*Populus balsamifera*	North America	Targeted genotyping	LFMM, Bayenv	GDM	Gougherty et al., 2021	Nature Climate change
*Quercus lobate*	United States	GBS	*F*_ST_ outlier test, LFMM	GF	Gugger et al., 2021	Molecular Ecology
*Quercus rugose*	Mexico	GBS	*F*_ST_ outlier test, LFMM	GF, GDM	Martins et al., 2018	Molecular Ecology
*Quercus* spp.	Switzerland	Poolseq	LFMM	RONA	Rellstab et al., 2016	Molecular Ecology
*Quercus suber*	Western Mediterranean	GBS	*F*_ST_ outlier test, SelEstim	RONA	Pina-Martins et al., 2019	Global Change Biology
*Betula nana*	United Kingdom	RADseq	*F*_ST_ outlier test, RDA, Bayenv2	RONA	Borrell et al., 2020	Evolutionary Applications
*Euptelea polyandra* and *Euptelea pleiosperma*	Japan and China	RAD	*F*_ST_ outlier test	GF	Cao et al., 2020	Evolutionary Applications
*Platycladus orientalis*	China	GBS	*F*_ST_ outlier test, Bayenv2	GF	Jia et al., 2020	Evolutionary Applications
*Quercus aquifolioides*	Western China	Poolseq	*F*_ST_ outlier test, Bayenv, LFMM	RONA	Du et al., 2020	Evolutionary Applications
*Pinus densata*	Western China	Exome capture sequencing	Bayenv, Pcadapt, RDA	GF	Zhao et al., 2020	New Phytologist
*Eucalyptus microcarpa*	Australia	DArTseq	*F*_ST_ outlier tests	RONA	Jordan et al., 2017	Molecular Ecology
*Corymbia calophylla*	Western Australia	DArTseq	Bayenv2, LFMM	GDM	Ahrens et al., 2019	Molecular Ecology
*Melaleuca rhaphiophylla* and *Nuytsia floribunda*	Southwestern Australia	DArTseq	*F*_ST_ outlier test, LFMM	GDM	Walters et al., 2020	Molecular Ecology

*DArTseq, diversity arrays technology sequencing; GBS, genotype-by-sequencing; GDM, generalized dissimilarity modeling; GF, gradient forest; GPA, genotype-phenotype association; LFMM, latent factor mixed model; Poolseq, whole-genome sequencing of pools of individuals; RADseq, restriction-site associated DNA sequencing; RDA, redundancy analysis; RONA, risk of non-adaptiveness.*

**TABLE 2:** Overview of methods and software available for environmental associations and genomic offset analyses in landscape genomics.

Software	Method	Purpose	Data type	Specifics and limitations	References
BAYENV, BAYPASS	Bayes	detecting GEAs	Allele frequencies and environmental variable	Less sensitive to population demography; but calibration with neutral SNPs is needed and significance thresholds need to be determined from simulated datasets.	Günther and Coop, 2013; Gautier, 2015
LFMM, R (LEA)	Bayes	detecting GEAs	Allele frequencies and environmental variable	Corrects for population structure using latent factors; but only performs association with environment.	Frichot et al., 2013; Frichot and Francois, 2015
SAMβADA, R (R.SamBada)	Spatial analysis	detecting GEAs	Allele frequencies and environmental variable	Underlying models are simple, allows correction for population structure; but possibly has high false-positive rates.	Stucki et al., 2017; Duruz et al., 2019
R (vegan)	Ordination	detecting GEAs	SNPs, environmental and geographic datasets	Finds the linear combinations of genetic and environmental datasets *via* RDA or CCA; but exists strong multicollinearity and doesn’t allow missing data.	XLSTAT, 2012; Oksanen et al., 2013
R (gdm)	GDM	projecting GF	Allele frequencies, environmental and geographic datasets	Provides genomic offset based on numbers of adaptive loci simultaneously *via* distance-based method; but result should be validated by additional datasets.	Manion et al., 2014; Fitzpatrick and Keller, 2015
R (gradientForest)	RF	projecting GF	Allele frequencies and environmental variables	Provides genomic offset based on numbers of adaptive loci simultaneously *via* machine-learning algorithm; but result should be validated by additional datasets.	Ellis et al., 2012; Fitzpatrick and Keller, 2015
pyRona	SLR	projecting GF	Allele frequency and environmental variable	Provides genomic offset based on average change in allele frequency at multiple adaptive loci; but result should be validated by additional datasets.	Rellstab et al., 2016; Pina-Martins et al., 2019

*CCA, canonical correlation analysis; GDM, generalized dissimilarity modeling; GEAs, genotype-environment associations; GF, genomic offset; RDA, redundancy analysis; RF, random forest; SLR, simple linear regression.*

## Existing Approaches of Landscape Genomics

### Mixed-Effects Models

Mixed-effects models provide a unified analytical framework to indicate robust and powerful evidence for adaptation (Rellstab et al., 2015). The advantage of the mixed-effects model is that it can reduce false-positive results by considering the influence of pairwise genetic distances and population structure. In mixed models, the genetic structure is incorporated as a random factor, allele frequencies are defined as response variables, and environmental factors are used as fixed factors. In this “Mixed-effects models” section, we illuminated the principles and methodologies using mixed models to detect signals of local adaptation based on BAYENV (Coop et al., 2010), Bayesian population association analysis (BayPass) (Gautier, 2015; Olazcuaga et al., 2020), latent factor mixed models (LFMMs) (Frichot et al., 2013), and spatial analysis method (SAM) (Joost et al., 2007, 2008).

The BAYENV is a method under the Bayesian framework employed to evaluate correlations between loci and environmental variables, and it can incorporate the uncertainty of allele frequencies from uneven sample sizes (Coop et al., 2010). The advantage of this program is that it applies a covariance matrix to take account for population structure, which is similar to an *F*_ST_ or kinship matrix. BAYENV requires a null model based on neutral loci and then determines the covariance matrix of estimated allele frequencies across populations. The significance test of each locus-variable combination utilizes Bayes factors calculated automatically by the program. However, cautions should be noticed here that these factors may not be directly compared across environmental variables due to variable-specific value ranges. In 2013, Günther and Coop developed an updated program called BAYENV2, which added non-parametric tests in the options and could be robustly applied for the Pool-Seq data (Günther and Coop, 2013).

BayPass (Gautier, 2015) is an extension of the Bayesian outlier detection model implemented in BAYENV to execute GEAs or environmental association analysis (EAA). It takes demographic effects into account by the estimation of the covariance matrix of allele frequency between populations. The core model (i.e., multivariate generalization model) in the BayPass reports locus XtX that is analogous to *F*_ST_ but explicitly corrected for this covariance matrix, accounting for the neutral correlations of allelic frequencies. Simulation studies suggested that the BayPass provided a robust framework to detect adaptive SNP signals (Gautier, 2015). However, a recent study revealed that the assumed linear relationships between allele frequencies utilized in EAA in line with the algorithm proposed by Gautier (2015) are unsatisfactory and even problematic when dealing with small datasets (Olazcuaga et al., 2020). Hence, they proposed a new approach that does not necessitate considering the uncertainty of the allele frequency estimation but assumes the exchangeability of SNPs both across the populations and along the genome. It is effective for gaining well-behaved *P*-values, avoiding intensively computational calibration, and providing reasonable numbers of SNPs analyzed (Olazcuaga et al., 2020). The advantage of BayPass is that it improves test performances by the estimations of the covariance matrix Ω (Olazcuaga et al., 2020).

The LFMMs rely on the Markov Chain Monte Carlo algorithms and integrate fixed effects to model environmental variables (Frichot et al., 2013). This algorithm is an extension of principal component analysis (PCA). LFMMs incorporate fixed effects to model environmental variables, and natural genetic structure is introduced as a random factor (i.e., latent factor). The computational speed is fast, and in addition, this approach does not need any *a priori* knowledge, making it attractive for determining adaptive signals with genomic data (Frichot et al., 2013; Rellstab et al., 2015). LFMMs can be implemented by the software LFMM (Frichot et al., 2013; Caye et al., 2019) or the R package *LEA* (Frichot and Francois, 2015).

The SAM is developed to assess putative associations between molecular markers and environmental variables using multiple univariate logistic regressions (Joost et al., 2007). It detects signatures of selection based on an integrative application of geographical information systems (GIS), environmental variables, and molecular data (Joost et al., 2007, 2008) implemented in MATLAB. The significance is determined by the likelihood ratio and Wald tests. Simulation studies implied that SAM might provide false-positive results if tested species endure complicated demography (De Mita et al., 2013; Frichot et al., 2013). Recently, an improved version of SAM called SAMβADA was developed (Stucki et al., 2017). This new approach allows for rapidly analyzing large genomics datasets by parallel processing. Compared with the early analysis method (i.e., SAM), the advantages of this new algorithm include that it (i) incorporates multivariate analyses to assess the impacts of many environmental predictor variables, (ii) allows to split the datasets and merges the results *via* parallel processing of SAMβADA, and (iii) enables the inclusion of explanatory variables representing population structure into the models to decrease false-positive results. However, pre- and post-processing of data will be labor-intensive when using the SAMβADA. In view of these facts, Duruz et al. (2019) published the R. SamBada landscape genomics pipeline to ease the identification and interpretation of candidate genes underlying local adaptation.

### Multivariate Statistical Analysis

The multivariate statistical analysis usually integrates environmental variables and spatial genetic structure into the analytical framework to detect the adaptive variation. Traditionally, isolation by environment (IBE) is commonly used to detect selection signatures (Wright and Gaut, 2004; Wang and Bradburd, 2014; Manthey and Moyle, 2015). However, this Mantel-based method had poor performance in detecting true-positive results (Harmon and Glor, 2010; Hardy and Pavoine, 2012; Legendre et al., 2015), and its estimation bias might be amplified in the genomic era. Instead, the multivariate statistical analysis such as canonical correlation analysis (CCA) (Ter Braak, 1986) and redundancy analysis (RDA) (Van Den Wollenberg, 1977; Legendre and Legendre, 2012) may be more realistic for detecting selection signatures than univariate methods (Forester et al., 2016, 2018), because the selection is always a polygenic process driven by multiple environmental factors.

The CCA aimed to find a linear relationship between multiple loci and environmental factors. The loadings consist of loci and environmental variables indicate which loci respond to which environmental factors. However, we need caution to infer the outcomes if strong patterns of multicollinearity exist within datasets (Rellstab et al., 2015; Fenderson et al., 2020). RDA is another ordination approach that is effective to detect adaptive variation based on allele frequency data (Legendre and Legendre, 2012; Capblancq et al., 2018). First, RDA produces a matrix of fitted values based on the multivariate linear regression between genetic and environmental data, and then, the PCA of the fitted values produces canonical axes that are linear combinations of the original explanatory variables (Forester et al., 2016). In addition, partial RDA (pRDA) that stems from RDA also allows for constructing and testing complicated models to avoid the impacts of neutral genetic structure or spatial effects on detecting loci underlying adaptive variation (Legendre and Legendre, 2012). Another analogous approach, called the distance-based redundancy analysis (dbRDA), which differs in associations between genetic data and principal coordinate analysis and the procedure of emerging response variables compared with the RDA, can also enable to detect the adaptive evolution (Legendre and Anderson, 1999). However, when using the abovementioned methods, an important caveat exists is that the explanatory variables within these methods are uncorrelated and the number of loci examined might be at least three times as large as the number of putative explanatory variables (Jombart et al., 2009).

Simulation and empirical studies suggested that the RDA-based method could detect lower false-positive and higher true-positive rates when compared with generalized linear models (GLM) or LFMM (Forester et al., 2018). Even the powers to identify adaptive loci associated with environmental variables are similar *via* RDA and LFMM, the former has the advantage to identify the main selective gradients as a combination of environmental variables (Capblancq et al., 2018). Additionally, the constrained ordination methods have robust performance and enable to avoid spurious GEAs when the tested species has isolation-by-distance (IBD) pattern or low dispersal capability (Forester et al., 2016, 2018; Capblancq et al., 2018).

## Predicting Genomic Vulnerability Under Alternative Climate Scenarios

Traditionally, the vulnerability of species mainly relied on the prediction of species distribution models (SDMs) (Elith and Leathwick, 2009) or its extensions, such as climate-niche factor analysis (CNFA) (Rinnan and Lawler, 2019). However, the abovementioned methods are unable to account for the continuous, multidimensional nature of genomic variation (Fitzpatrick and Keller, 2015). The expanding omics and statistical tools enable us to generate robust predictions of plant adaptive potential under climate change. In this study, we introduced three new methods used for predicting genomic vulnerability under alternative climate scenarios based on the linear or non-linear functions: generalized dissimilarity modeling (GDM) (Ferrier et al., 2007), gradient forests (GFs) (Ellis et al., 2012), and RONA (Rellstab et al., 2016).

### Predicting Genomic Vulnerability Using Non-linear Regressions

**FIGURE 2:**
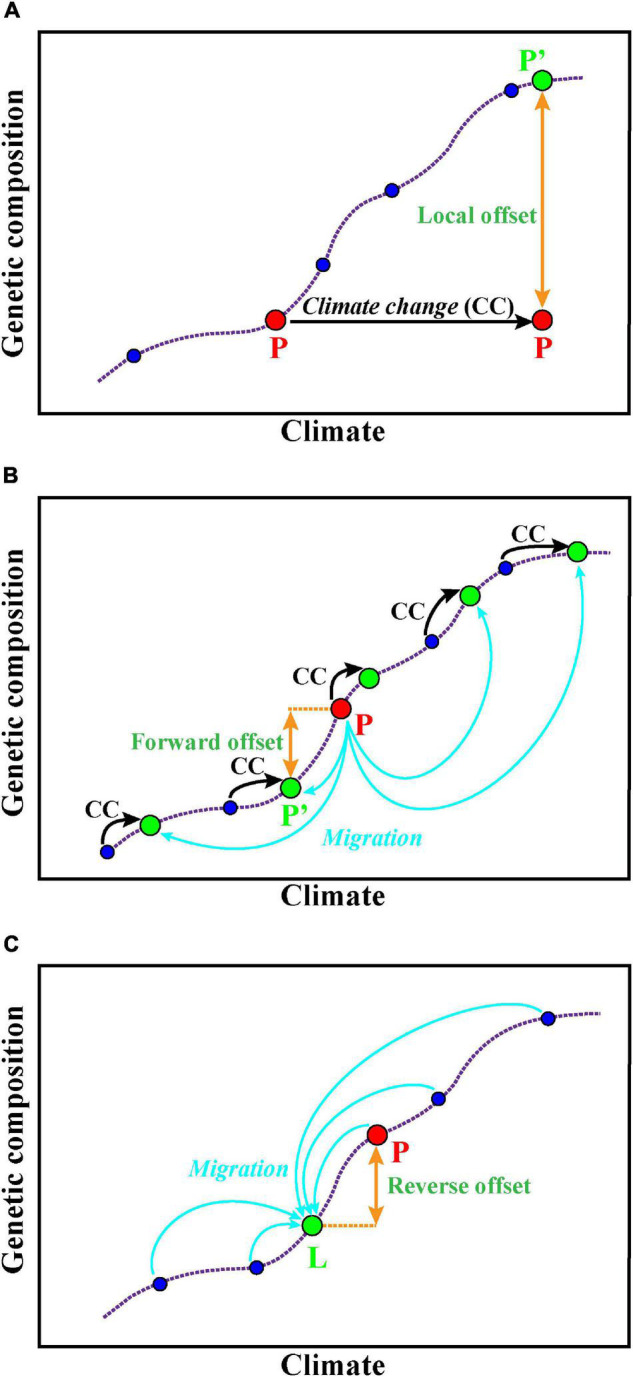
Predictions of potential adaptation to alternative climate scenarios. **(A)** Local offset means the specific population P (red color) to the theoretically required changes of allele frequency under a future climate *in situ* [P′ (green)]. **(B)** Forward genetic offset means that a specific contemporary population P (red color) can migrate (blue arrows) to the habitat whose future climate best matches its genetic composition [P′ (green)]. **(C)** Reverse genetic offset means for a specific location L (green color) and its future climate, the minimum genetic distance of a contemporary population P (red color) to the theoretically required population for location L. The purple dotted line represents the association between the genetic composition of several populations (blue circles) and their local, contemporary climate. These figures are modified from Rellstab (2021).

The GDM is used for estimating and predicting the spatial pattern of turnover in community composition (Ferrier et al., 2002, 2007). Fitzpatrick and Keller (2015) extended the application of GDM to forecast the genetic offset of *Populus balsamifera*, and they concluded that the changes of genetic composition are required if it tries to mitigate maladaptation and maintain genetic diversity in the future. GDM accounts for spatial patterns in genetic data caused by demographic processes, accommodates varied factors (e.g., geographic or ecological separation, barriers to dispersal) as predictors, and also enables to deal with numerous SNP loci (Fitzpatrick and Keller, 2015). The functions can be implemented *via* the R package *gdm* (Manion et al., 2014). A recent study evaluated the local, forward, and reverse genetic offsets (Figure 2) of balsam poplar using the GDM and incorporated migration and dispersal into predictive genomic models to show the adaptive potential of balsam poplar in future climates (Gougherty et al., 2021). This study provides a new way to assess population-level risk at alternative climate scenarios that accounts for local adaptation and breaks through the prediction limitations at the species level.

The GF is an extension of random forests based on the non-parametric, machine-learning regression tree approach (Ellis et al., 2012). This method enables to estimate and map the frequency changes of SNPs associated with environmental tolerance at different spatial-temporal scales (Fitzpatrick and Keller, 2015). It can be executed *via* the R package *gradientForest* (Ellis et al., 2012). Unlike GDM, GF can handle complicated associations between predictors and accommodate these correlated predictors, providing a means to determine the response of individual SNPs to environmental gradients.

Both GDM and GF can handle large genomic datasets that include numerous rare alleles and accommodate pronounced non-linearities in the exploration of GEAs, providing unprecedented insights into genome regions under local selection and predicting the changes of adaptive genomic diversity across landscape. For instance, Martins et al. (2018) revealed a strong association between the genetic variation of *Quercus rugosa* and the precipitation seasonality in Mexico *via* the GDM and GF, and they predicted that future populations of *Q. rugosa* might be at risk due to the high rate of climate change. However, considering that the actual evolutionary responses of populations to climate change will be more complex than the simplified projections based on the two abovementioned approaches, we must consider caveats when explaining the result of genetic offset arising from the GDM and GF.

### Predicting Genomic Vulnerability Using Linear Regression

**FIGURE 3:**
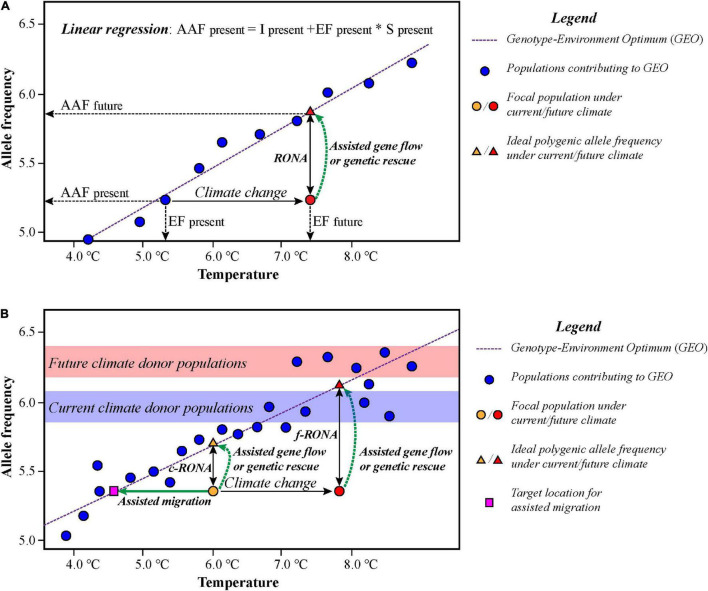
Schematic illustration of the risk of non-adaptedness (RONA) to alternative climate change. **(A)** RONA is the average change of allele frequency in a set of adaptive loci that are required under future climate scenario according to a simple linear regression of the relationship of allele frequency and environments. AAF, alternative allele frequency; EF, environmental factor; I, intercept of the regression; S, slope of the regression. **(B)** The current and future RONA (c-RONA and f-RONA); c-RONA/f-RONA is the average change in allele frequency required under current environmental conditions. Blue and red bands indicate suitable candidate donor populations for assisted gene flow under current and future scenarios, respectively. The figures **(A,B)** are modified from Rellstab et al. (2016) and Borrell et al. (2020), respectively.

Rellstab et al. (2016) developed a method called RONA to evaluate genomic vulnerabilities of populations under alternative climate scenarios based on linear regressions inspired by the study of assessing the relative risk of maladaptation in Douglas fir (Bradley St Clair and Howe, 2007). RONA represents the average change in allele frequency at adaptive SNPs required to keep pace with the change of a given environmental factor in future (Figure 3). The average absolute difference of the changes in allele frequencies of these loci between the current and future climate conditions represents RONA under a given environmental variable (Rellstab et al., 2016; Pina-Martins et al., 2019). Recently, Borrell et al. (2020) utilized the current RONA (c-RONA, Figure 3) to define the average change in allele frequency at climate-associated loci required to match the estimation of the optimum for a given environmental factor, using the future RONA (f-RONA; Figure 3) to define the original concept of RONA proposed by Rellstab et al. (2016).

Theoretically, if the difference between the current and the prediction values is high, then more conservation efforts are needed for persisting of focal species. Empirical studies in trees and invertebrates show that if the expected allele frequency changes are less than 0.1 per decade, it might keep pace with climate change, while if the changes are greater than 0.1–0.2 per decade, it may cause a lag between allele frequency and climate adaptation (Jump, 2006; Egan et al., 2015; Jump et al., 2017). However, this simplified approach does not take gene flow and migration into account and assumes that the best model profiling the GEAs is resulted from local adaptation. Furthermore, the predictions of RONA for adaptive loci based on this method have multiple values, and each RONA arises from a given environmental variable, which will not account for the interactive effects of loci contributing to climate adaptation (Rellstab et al., 2016; Capblancq et al., 2020). Therefore, we must keep in mind that the genomic vulnerability approaches are still in their infancy and face numerous challenges and uncertainties, and they have yet to be tested and validated in real conservation applications (Rellstab, 2021).

## Applications of Landscape Genomics in Tree Conservation

Landscape genomics significantly improves our understanding of ecological and evolutionary processes in tree species and offers guidelines for conservation efforts and management applications. The potential of landscape genomics for forest management is discussed in the following sections.

### Using Landscape Genomics to Inform Genetic Rescue

Genetic rescue aims to increase population fitness and avoid population declines by introducing immigration of new alleles (Tallmon et al., 2004; Whiteley et al., 2015; Bell et al., 2019; Fitzpatrick and Funk, 2021). Landscape genomics studies will increase the effectiveness of genetic rescue by identifying which populations are most likely to increase fitness and population growth rate (Whiteley et al., 2015). The population with the lowest level of adaptive differentiation would be chosen in order to minimize outbreeding impression. A recent study on dwarf birch suggested that the genetic rescue should be applied for the populations with small population sizes that occurred in the margins of the distribution of species (Borrell et al., 2020). However, previous studies had indicated that genetic rescue can only improve fitness and increase population sizes in the short term rather than save imperiled populations over the long term (Whiteley et al., 2015). Additionally, if the inbreeding depression in small populations resulted from the recent effect of human-caused fragmentation, assisted migration is more appropriate than genetic rescue (Hohenlohe et al., 2021).

### Using Landscape Genomics to Inform Assisted Gene Flow

Assisted gene flow (AGF) means managed translocation of individuals within the current species range to mitigate local maladaptation (Aitken and Whitlock, 2013; Aitken and Bemmels, 2016). AGF is equivalent to the genetic rescue when target populations are small and maladapted, with genetic diversity therein dominantly threatened by drift (Aitken and Whitlock, 2013). Compared with genetic rescue, AGF emphasizes the introduced alleles that are preadapted to new local environments and thus increase the frequency of these adaptive loci in existing populations. AGF has already been applied for some forest trees. For example, Browne et al. (2019) suggested that AGF might be applied to mitigate adaptation lag of temperature for California oak according to a landscape genomic survey of the species. Another fascinating case is dissecting the associations of GEAs in balsam poplar (Gougherty et al., 2021). Gougherty et al. found that the eastern populations of the balsam poplar might face the greatest vulnerability and risk of future extirpation to climate change, and the conservation efforts *via* AGF are needed for those populations by estimating the local, forward, and reverse genetic offsets of the species. However, outbreeding depression might occur if source and recipient populations are isolated for a long time (Aitken and Whitlock, 2013). Additionally, high levels of gene flow introduced by AGF might result in biotic homogenization between source and target populations and consequently prevent them from adapting to novel climate conditions (Gaitán-Espitia and Hobday, 2021).

### Using Landscape Genomics to Inform Seed Sourcing Strategy

Seed sourcing strategy aims to capture the adaptive diversity and improve the adaptive potential of species under climate change and has been proposed for ecological restoration during past decades (Broadhurst et al., 2008; Breed et al., 2013, 2019). Landscape genomics is an ideal approach to inform seed sourcing strategies for species persisting. Jordan et al. (2017) detected 81 putatively adaptive SNPs in *Eucalyptus microcarpa*, and 62 of which are associated with mean annual temperature by a combination of four *F*_ST_ outlier tests and one EAA (i.e., BAYENV2) as the general framework of landscape genomics (Figure 1). They found that the expected allelic frequency changes of these adaptive SNPs in the New South Wales (NSW) populations were greater than that of other sites, suggesting that the warmer, northern end of the range (i.e., NSW) of *E. microcarpa* might not suitable for seed source. Recently, a provenance decision-making framework proposed by Carvalho et al. (2021) offers a comprehensive perspective for seed source guidelines based on the information that arises from neutral and adaptive variation *via* integrative analyses of population genomics and landscape genomics, which can also be applied for informing seed sourcing strategy of forest species.

## Challenges and Future Directions

Landscape genomics provides unprecedented insights into understanding the mechanism of adaptive variation of tree species by dissecting the impacts of environmental variables and landscape characteristics on their adaptive evolution (Browne et al., 2019; Pina-Martins et al., 2019; Borrell et al., 2020; Du et al., 2020; Zhao et al., 2020). Common challenges, such as the sampling strategies and using only a single analysis for detecting adaptive signatures, still exist in landscape genomic studies although many reviews discuss the abovementioned topics (Balkenhol et al., 2019). Instead of discussing the abovementioned common challenges in this study, we focused on the challenge of landscape genomics studies on tree species in identifying adaptive variation and their spatial patterns facing the changing climate.

First, the levels of commonality in genes or SNPs associated with climates that arose in landscape genomics studies are quite low. Although the large majority of landscape genomics studies utilize integrative methods for detecting putatively adaptive loci to illuminate the GEAs, few loci are shared between these approaches. These inconsistent patterns by different methods were detected in many studies, for example, in Mexican oak *Q. rugose* (Martins et al., 2018: only one SNP associated with temperature seasonality was identical between LFMM and BAYESCAN test) or Norway spruce in three independent landscape genomic studies across the Italian Alps sharing similar sampling areas and climates (Scalfi et al., 2014; Ćalić, 2015; Di Pierro et al., 2016: no identical adaptive genes were detected in more than two studies).

The low commonality in adaptive signals might be the evidence for lacking parallel evolution of adaptive traits in forest trees (Geraldes et al., 2014) or just because of false-positive results (Ćalić et al., 2015). Additionally, we believed that the varied methods applied for detecting adaptive SNPs have different assumptions, advantages, and limitations, which responded to the low commonality. However, the commonality levels for projecting genomic vulnerability under alternative climate scenarios using GF or GDM are relatively high. Fitzpatrick and Keller (2015) found that using GDM and GF for the projections of genomic vulnerability, the genetic offset of the circadian clock gene GIGANTEA-5 (*GI5*) associated with plant circadian clock and light perception pathways in balsam poplar is similar, although slight differences existed in its marginal area, both methods predict the range core of balsam poplar likely suffered minimal disruption of the existing GEAs. In the future, efforts by a combination of simulations, genomic data, and common garden experiments might be applied to demonstrate the high effectivity and accuracy of genomic offset under alternative climates (Fitzpatrick et al., 2021).

Second, the current studies of landscape genomics for evaluating and uncovering the adaptive variation in tree species focus only on a single species rather than at the community level (Table 1). Analyzing multiple species within the same landscapes makes it possible to assess the commonality of their eco-evolutionary dynamics across species and landscapes and thereby depict a thorough picture of how local adaptation is originated in nature (Bragg et al., 2015; Hand et al., 2015; Balkenhol et al., 2019). However, eco-evolutionary models require new data and methods for assessing the adaptive potential of species, which have only been possible for a few model species so far (Waldvogel et al., 2020). In addition, the present challenge of illuminating ecological adaptation at the community level is how to simulate the patterns of local adaptation of species or populations and their adaptive potential under future climate changes, while a possible way to overcome these inconveniences is integrating the prediction methods including GDM, GF, or RONA into the analytical framework of landscape community genomics.

Finally, the investigators of landscape genomics must consider the genomic sequencing strategy employed and the genomic resources available for their focal species. Prevalent sequencing methods in the landscape genomics studies of non-model species currently take advantage of reduced-representation methods [e.g., genotype-by-sequencing (GBS) and restriction-site associated DNA sequencing (RADseq)] and RNA sequencing. However, the number of SNPs obtained and the ability to detect genes underlying local adaptation from the abovementioned methods may be influenced due to the differences in library preparation, SNP densities, and the bioinformatics parameters applied to SNP filtering (Hoban et al., 2016; Lowry et al., 2017; McKinney et al., 2017). As more and more forest tree genomes have been published (e.g., Table 1 in Ingvarsson et al., 2016) and sequencing costs fall, whole-genome resequencing is thriving and becoming an option for landscape genomics studies (Lin et al., 2018; Zhu et al., 2020), which can provide unprecedented marker density and determine other genetic variation such as structural variants and mutations in regulatory elements, increasing power for the detection of local adaptation and providing novel insights into the role of selection, recombination, and gene flow in promoting or impairing local adaptation to new habitats compared with reduced-representation methods (Fuentes-Pardo and Ruzzante, 2017; Bourgeois and Warren, 2021). In addition, the degrees of linkage disequilibrium (LD) in the studied species will also influence the power of detecting adaptive SNPs. Considering the low LD rates in tree species, using these methods such as reduced-representation methods will fail to detect loci that underlie most local adaptation and adaptive phenotypic variation (Bragg et al., 2015; Hoban et al., 2016). We advocated obtaining detailed LD information of focal species using whole-genome sequencing before the studies of landscape genomics in future because the resources of reference genome are critical to fully address the issues of local adaptation (Manel et al., 2016). Moreover, prior knowledge about LD decay from the reference genome of focal species can inform sampling strategies and sequencing selections to maximize opportunities to identify adaptive SNPs (Bragg et al., 2015).

## Conclusion

Understanding the genetic mechanism of adaptation is the key issue for molecular ecology and evolutionary biology. We reviewed the existing theories and methods that belong to landscape genomics for detecting adaptive evolution in species and advocated utilizing an integrated analytical framework to illuminate the GEAs between genetic and environmental data. We particularly emphasized the effectivity and necessity of multiple methods for detecting signatures of local adaptation combined with models for predicting adaptation potential in tree conservation. With the low sequencing cost, ease availability of high-solution environmental data, and newly developed genomic tools in the near future, we believe that the conservation efforts and management interventions for forest trees will benefit from advancing studies of landscape genomics.

## Author Contributions

LF and FD conceived the study and wrote the manuscript. Both authors designed the focus, structure and content of the review.

## Conflict of Interest

The authors declare that the research was conducted in the absence of any commercial or financial relationships that could be construed as a potential conflict of interest.

## Publisher’s Note

All claims expressed in this article are solely those of the authors and do not necessarily represent those of their affiliated organizations, or those of the publisher, the editors and the reviewers. Any product that may be evaluated in this article, or claim that may be made by its manufacturer, is not guaranteed or endorsed by the publisher.
