# Corrigendum: Landscape Genomics in Tree Conservation Under a Changing Environment

**DOI:** 10.3389/fpls.2022.911163

**Published:** 2022-04-28

**Authors:** Li Feng, Fang K. Du

**Affiliations:** ^1^School of Pharmacy, Xi'an Jiaotong University, Xi'an, China; ^2^School of Ecology and Nature Conservation, Beijing Forestry University, Beijing, China

**Keywords:** changing environment, genotype-environment associations (GEAs), landscape genomics, local adaptation, tree conservation

In the original article, there was a mistake in the legend for Figure 1 titled as **The general framework of landscape genomics for tree conservation** as published. We missed the proper citations about plots of *F*_ST_ outlier test, cluster and RONA which were depicted in Figure 1. The correct legend appears below.

**Figure 1:**
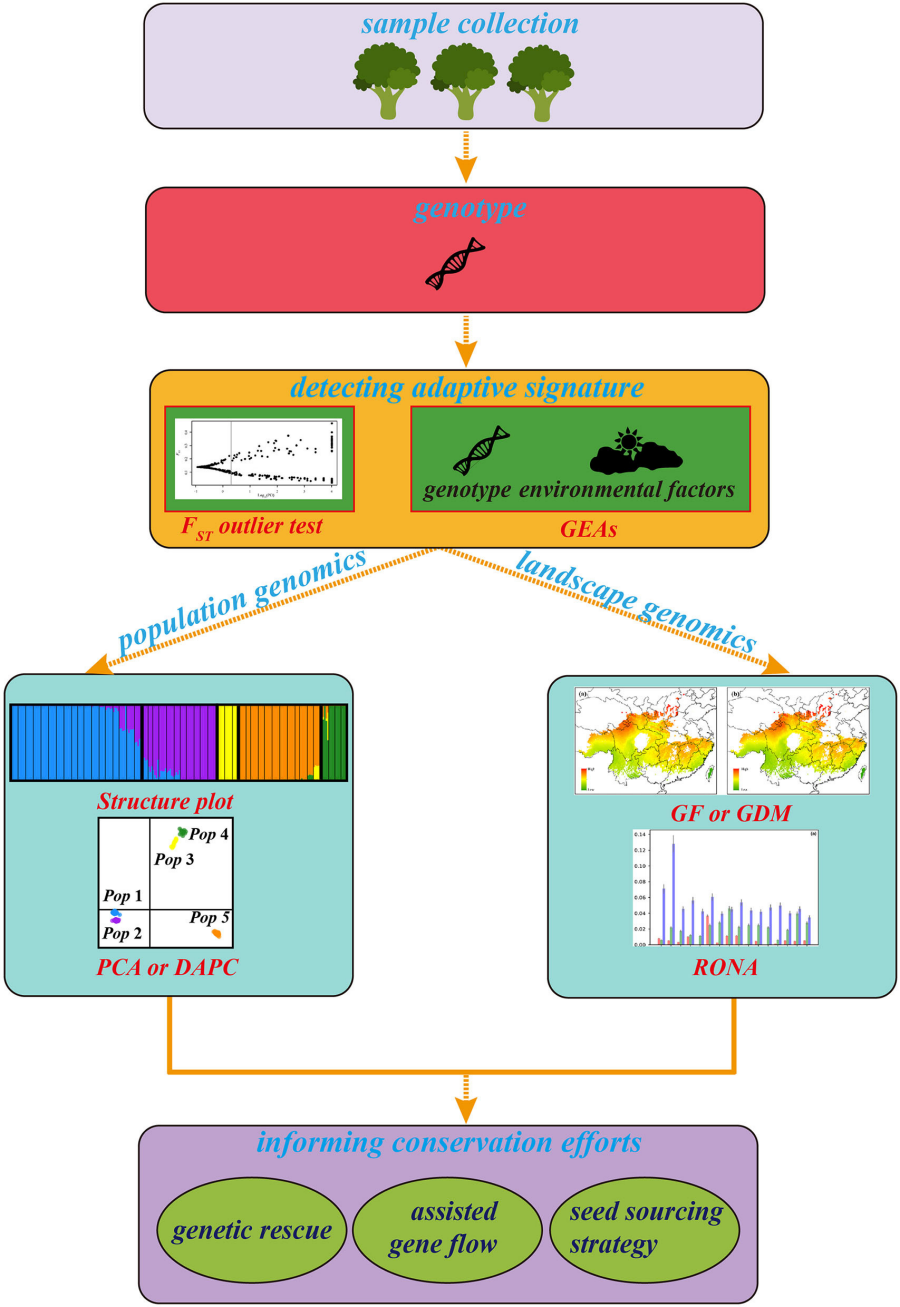
The general framework of landscape genomics for tree conservation. The plots of cluster, *F*_ST_ outlier test and RONA are modified from Du et al. (2020) and Feng et al. (2020), respectively.

In the original article, there was a mistake in Figure 1 as published. “In the early Figure 1 of this article, it contained some elements that debate might arise.” The corrected Figure 1 appears below.

In the original article Feng, L., Ruhsam, M., Wang, Y. H., Li, Z. H., and Wang, X. M. (2020). Using demographic model selection to untangle allopatric divergence and diversification mechanisms in the *Rheum palmatum* complex in the Eastern Asiatic Region. *Mol. Ecol*. 29, 1791-1805. doi: 10.1111/mec.15448 was not cited in the article. The citation has now been inserted in the legend of Figure 1 and should read:

The general framework of landscape genomics for tree conservation. The plots of cluster, *F*_ST_ outlier test and RONA are modified from Du et al. (2020) and Feng et al. (2020), respectively.

The authors apologize for this error and state that this does not change the scientific conclusions of the article in any way. The original article has been updated.

## Publisher's Note

All claims expressed in this article are solely those of the authors and do not necessarily represent those of their affiliated organizations, or those of the publisher, the editors and the reviewers. Any product that may be evaluated in this article, or claim that may be made by its manufacturer, is not guaranteed or endorsed by the publisher.
